# Studies on the changes in rectal permeability and intestinal microbiota with developmental age in young rats

**DOI:** 10.3389/fmicb.2025.1551693

**Published:** 2025-04-17

**Authors:** Yunfeng Luo, Liangming Luo, Mengle Xia, Qian Liu, Guosong Zhang

**Affiliations:** ^1^Discipline of Chinese and Western Integrative Medicine, Jiangxi University of Chinese Medicine, Nanchang, China; ^2^Jiangxi University of Chinese Medicine, Nanchang, China; ^3^Yudu County Hospital of Traditional Chinese Medicine, Ganzhou, China

**Keywords:** early development, rectum, permeability, microbiota, tight junction proteins

## Abstract

**Introduction:**

The gut contains a diverse array of commensal microorganisms, forming a vital biological barrier within the intestine that contributes to the overall intestinal mucosal barrier. However, research on the rectal barrier during early development remains limited. This study aims to investigate the relationship between intestinal microbiota and rectal barrier function in young rats.

**Methods:**

We evaluated the rectal barrier structure and function in rats at 2-, 4-, and 10-week-old. Methodology included histological analysis, Muc2 expression quantification, immunofluorescence localization of tight junction proteins (ZO-1, Occludin, Claudins), blood glucose monitoring after rectal insulin administration, and 16S rDNA sequencing of rectal microbiota. Spearman correlation analysis was used to explore mechanisms linking age-dependent changes in rectal permeability to microbiota dynamics.

**Results:**

Physiological rectal permeability was significantly higher in 2-week-old rats compared to 4- and 10-week-old rats (*p* < 0.01), although systemic biomarkers (LPS, D-LA, and LBP) showed no significant differences. The rectal microbiota exhibited marked age-dependent shifts in composition, *α*/*β*-diversity, and metabolic pathways, with increased abundance of beneficial taxa (e.g., Muribaculaceae, Akkermansia) in older rats. Correlation analysis revealed strong associations between reduced permeability, elevated Occludin expression, and microbiota maturation (*R* = 0.65, *p* < 0.001).

**Conclusion:**

This study demonstrates that age-dependent maturation of the rectal barrier is closely linked to microbiota composition and tight junction protein expression, providing insights into developmental mechanisms and potential strategies for pediatric rectal drug delivery.

## 1 Introduction

Oral medication is the most common route of administration for patients. For pediatric populations, the rectal route is an excellent alternative, as it does not require swallowing and is less strenuous. This method is also applicable in emergency situations for children who are unconscious or vomiting. When studying intestinal drug absorption, assessing intestinal permeability is essential (Dahlgren and Lennernäs, 2019). However, most knowledge about drug absorption is based on oral administration to the small intestine, especially in adults, which overlooks the immature nature of the rectum in early life stages.

Intestinal permeability is primarily governed by tight junction proteins located within the intercellular spaces, which restrict the passage of molecules larger than 600 Da (Shen et al., 2011). Osmotic enhancers are often added to rectally administered formulations to enlarge these intercellular gaps, thereby increasing drug absorption (Luo et al., 2021). young rats express lower levels of tight junction proteins compared to adults (Gleeson et al., 2021), but it is not clear whether the change of rectal permeability is related to age.

Furthermore, the colonization of the mammalian gut by microbial populations plays a crucial role in regulating body physiology, metabolism, and immunity. Microbial colonization begins in the womb and continues after birth until a stable intestinal microbial environment is established (Wopereis et al., 2014). This colonization maintains the integrity of the intestinal epithelial barrier by altering the transcriptomic profile of intestinal epithelial cells and promoting their proliferation and differentiation (Kerr et al., 2015). Bacterial structures within the gut can regulate intestinal barrier function through the activation of Toll-like receptors (TLRs) and NOD-like receptors (NLRs) pathways (Ghosh et al., 2021). Additionally, metabolites produced by the microbiota can exert regulatory effects on the intestinal mechanical barrier, with short-chain fatty acids (SCFAs) and bile acids being key products. It has been shown that butyrate can increase the expression of tightly junction proteins, such as Occludin and ZO-1, in IPEC-J2 cells and reduce intestinal permeability in rats (Wang et al., 2012). Tong et al. found that propionate can enhance the expression of tight junction proteins Occludin and ZO-1, as well as calcineurin (Tong et al., 2016).

The intestinal flora exhibits distinct characteristics across different segments of the gastrointestinal tract (Zhao et al., 2015). Currently, studies on intestinal flora are typically conducted through fecal testing, which does not accurately reflect the characteristics of specific intestinal segments (Tang et al., 2020). To address this, we analyzed the characteristics of the rectal mucosal microbiota using rectal swab sampling, aiming to uncover age-related changes from young to adulthood. We investigated the changes in rectal permeability as rats develop from young through different stages to adulthood. This included assessing the mechanical barrier function of the rectum, which consists of the intestinal epithelial barrier and the mucus barrier, at various developmental stages. Our methodology involved histological analyses of the rectum, measurement of mucin expression levels, evaluation of the expression and localization of tight junction proteins, and monitoring glucose levels in rats with rectally administered insulin to assess functional insulin absorption. Additionally, we analyzed the rectal microbiota using 16S rDNA sequencing. Finally, we conducted Spearman’s correlation analysis to determine the relationship between rectal barrier function and the rectal microbiota.

## 2 Materials and methods

### 2.1 Materials

FITC-Dextran (Sigma), all antibodies, including ZO-1 antibody, Occludin antibody, Claudin-1 antibody, and goat IgG H&L antibody (Abcam, UK), D-lactate acid (D-LA) detection kit (Megazyme), all primers (Sangon Biotech), lipopolysaccharide (LPS) detection kit (Wuhan Huamei), lipopolysaccharide binding protein ELISA Kit (Biomatik, China), qRT-PCR reagents and kits (Novozymes, Nanjing, China), bovine pancreatic insulin (Sigma-Aldrich), as well as blood glucose test strips and a blood glucose monitor (Roche), among other items.

### 2.2 Experimental animals

The operations involving animals in this experiment strictly adhered to the relevant provisions set forth by the Animal Research Committee of Jiangxi University of Traditional Chinese Medicine. The experimental program was reviewed and approved by the Jiangzhong Experimental Animal Ethics Committee (Ethics No. BCTG-2016-18). The animals used in this study were Sprague Dawley (SD) rats. Neonatal rat litters, along with their mothers (12–14 rats per litter), were purchased from Hunan Slaughter Kingdard Company. The rats were maintained in a clean-grade laboratory with a constant temperature of 22°C-25°C and humidity levels of 45–55%. They were housed in a pathogen-free environment under controlled humidity and temperature conditions, with a 12:12 h light/dark cycle, and had access to adequate laboratory food and filtered water. The rats were weaned at 3 weeks of age, after which males and females were housed in separate cages. During sampling, the entire rectum and blood were collected as detailed in the following sections.

### 2.3 FITC-dextran based assay for rectal permeability

The experimental animals were assessed for rectal permeability at the second, fourth, and tenth weeks of age. Prior to the experiment, they were fasted overnight and weighed to facilitate the emptying of rectal feces. Each rat was then anesthetized with isoflurane, and a horizontal incision was made in the abdomen to locate the colorectum. If feces remained in the rectum, a saline enema was administered to assist in emptying it. The upper end of the rectum was ligated carefully to avoid damaging any mesenteric vessels. FITC-Dextran (200 mg/kg, 100 mg/mL) was subsequently injected from the anus using a 1 mL gastric perfusion needle, and the anus was sealed with a biological adhesive to prevent leakage. Blood samples were drawn at 1 h and 3 h post-injection, and plasma was separated. The fluorescence signal intensity was measured using a multifunctional enzyme-labeled instrument at an excitation/emission wavelength of 485 nm/525 nm. A standard curve was drawn to calculate the concentration of FITC-Dextran. Rectal permeability was evaluated based on the plasma FITC-Dextran level measured within the 3-h window.

### 2.4 Detection of intestinal permeability markers: LPS, D-LA and LBP

In this phase of the experiment, blood was collected from the orbital vein, and plasma was separated. Plasma levels of LPS, D-LA and LBP were detected using the ELISA method, following the instructions provided with the respective kits.

### 2.5 Histological analysis of rectum

At the conclusion of the experiment, we euthanized the rats and carefully retrieved their rectal tissues. We then trimmed the tissue blocks to the appropriate size and placed them in Carnoy’s fixative. Following this, we embedded the tissues in paraffin wax through a process involving gradient ethanol dehydration and xylene transparency. Next, we sliced the embedded tissue and gradually removed the wax using a gradient of xylene, followed by rehydration with a gradient ethanol solution at 60°C for 2 h. The tissue sections were then stained with hematoxylin and eosin (HE) and Alcian Blue-Periodic Acid Schiff (AB-PAS) stains. Finally, we measured the depth of the rectal crypts and manually counted the number of goblet cells within the crypts per unit area using ImageJ software (NIH, Maryland, USA).

### 2.6 Immunofluorescence staining

We first dewaxed and hydrated the paraffin sections, then performed antigenic heat repair using sodium citrate buffer (pH 6) in a microwave oven. Next, we sealed the sections with goat serum. The primary antibody, Occludin antibody at a dilution of 1:200, was incubated overnight at 4°C in a humidity-controlled box. Following this, the sections were incubated with FITC-labeled goat anti-rabbit secondary antibody at a dilution of 1:200 for 1 h at room temperature, protected from light. Afterward, DAPI was applied for 10 min, also away from light, and the anti-fluorescence coverslip was sealed with an anti-fluorescence quencher. Finally, the images were observed and captured using a fluorescence microscope.

### 2.7 Quantitative real-time fluorescence quantitative PCR analysis

Tissue samples were placed in 0.5 mL of Lysis Buffer and homogenized using a tissue breaker. Next, 0.1 mL of chloroform was added, and the mixture was centrifuged at 12,000 rpm for 2 min. The supernatant was carefully aspirated and mixed with an equal volume of 100% ethanol, then centrifuged at 4,000 rpm and 4°C for 1 min. Following this, 500 μL of Wash Buffer was added, and the mixture was centrifuged at 12,000 rpm for 1 min at 4°C three times. The resulting solution was transferred to a new centrifuge tube, and 30 μL of Elution Buffer was added to the center of the column. This was allowed to sit for 2 min before being centrifuged at 12,000 rpm for 1 min at 4°C to elute the RNA. RNA was extracted using the Tissue RNA Extraction Kit (Eisenbach Bio RN002plus). The HiScript II qRT SuperMix for qPCR (username_1 DNA wiper) Reverse Transcription Kit (Nanjing Novizen, R223-01) was then used to generate cDNA from 1 μg of RNA. The cDNA synthesis was performed using a StepOne Plus fluorescence quantification instrument. Quantitative real-time polymerase chain reaction (qRT-PCR) was conducted on the same instrument using 2 × ChamQ Universal SYBR qPCR MasterMix (Vazyme). Primer sequences are detailed in Table 1.

**Table 1:** PCR primer sequence.

Gene	Direction	Primer sequence (5’→3’)
TNFα	Forward	*GCCTTGCCTTGCTGCTCTACC*
	Reverse	*CTTCGTGGGGTGTGTGTGCTCTCC*
IL-6	Forward	*ACTTCCAGCCAGTTGCCTTCTTG*
	Reverse	*TGGTCTGTGTGTGGGTGGTTCCTC*
ZO-1	Forward	*ACCCGAAACTGATGCTGTGGATAG*
	Reverse	*AAATGGCCGGGCAGAACTTGTGTA*
Occludin	Forward	*ACGGACCCTGACCACTATGA*
	Reverse	*TCAGCAGCAGCCATGTACTC*
Claudin-1	Forward	*AGCTGCCTGTTCCATGTACT*
	Reverse	*CTCCCATTTGTCTGCTGCTC*
Claudin-2	Forward	*CCTCCTGGCTGAGACTCCATCACCTT*
	Reverse	*GTGCTGACGATAGAGCCGATCCATCC*
Muc2	Forward	*GCTGACGAGTGGTTGGTGAATG*
	Reverse	*GATGAGGTGGCAGACAGGAGAC*
Claudin-3	Forward	*ATTCATCGGCAGCAGCATCATC*
	Reverse	*CAGCAGCGAGTCGTACATCTTG*
IL-1β	Forward	*AATCTCACAGCAGCATCTCGACAAG*
	Reverse	*TCCACGGGCAAGACATAGGTAGC*
Gapdh	Forward	*GCGAAAGCATTTGCCAAGAA*
	Reverse	*GGCATCGTTTATGGTCGGAAC*

### 2.8 Rectal insulin administration test

The experimental animals were fasted overnight and weighed prior to the experiment. They were then assisted in emptying their rectal feces and anesthetized with isoflurane. A horizontal incision was made in their abdomen to access the colorectum. If feces remained in the rectum, a saline enema was administered to aid in its evacuation. The upper end of the rectum was ligated carefully to avoid damaging the mesenteric blood vessels. A 1 mL gavage needle was used to deliver a bovine insulin solution (5 U/kg, 5 U/mL) into the rectum. The rectum was subsequently sealed with a biological adhesive to prevent leakage, and the anus was also secured with the adhesive. Blood glucose levels were measured from tail-tip blood samples at 0.5, 1, 1.5, 2, and 3 h post-administration, with a saline solution rectal administration group serving as the control.

### 2.9 Rectal swabs, DNA extraction and 16 s gene based analysis

Rectal swab samples were collected in a bacterial holding solution and stored at −80°C. Genomic DNA was extracted from these rectal swab samples using a fecal DNA extraction kit that employs Phusion High-Fidelity PCR Master Mix. The concentration and purity of the DNA were assessed through 2% agarose gel electrophoresis, and the samples were diluted for future use. To amplify the V4 region of the bacterial 16S rRNA gene, we utilized specific primers: the forward primer 515F (*5‘-GTGCCAGCMGCCGCGGGTAA-3’*) and the reverse primer 806R (*5′ - GGACTACHVGGGTAA-3′*). The resulting PCR products were purified using AMPure XP magnetic beads and quantified with the PicoGreen dsDNA Assay Kit (Invitrogen, Carlsbad, CA, USA). Following purification and quantification, high-throughput sequencing was performed using an Agilent 2,100 Bioanalyzer and the Illumina NovaSeq 6,000 platform. Valid sequences were obtained through quality control of the raw data using Qiime II and Trimmomatic. Alpha and beta diversity analyses were conducted on the 16S rDNA sequencing feature list and its corresponding feature sequences. The alpha diversity analysis was used to evaluate the diversity and richness of the environmental samples, incorporating indices such as Shannon, Simpson, and Chao1 (Hill, 1973). In contrast, beta diversity was primarily employed to cluster samples through Principal Coordinate Analysis (PCoA). Rat rectal swab samples were identified based on the ASV (feature) table and the NT-16S database, with species being labeled and counted. To compare multiple groups of samples with biological replicates, the Kruskal-Wallis test was utilized to assess differences between the comparison groups. Graphs were generated using the R programming language.

### 2.10 Statistical methods

GraphPad Prism 9.5 software was utilized for statistical analysis. One-way ANOVA was employed to compare data samples from multiple groups, while the independent *t*-test was used for comparisons between two independent groups. The Wilcoxon signed-rank test was applied to assess measurement data between two groups of microbiota samples. For comparisons involving multiple samples with biological replicates, the Kruskal-Wallis test was used, and count data were analyzed using the chi-square test. The Spearman correlation test was conducted to examine the association between rectal barrier function and microbiota development. Data are presented as mean ± standard deviation. *T*-tests and one-way ANOVA analyses were performed using GraphPad Prism software, with statistical significance indicated as follows: *p ≤ 0.05; **p ≤ 0.01; ***p ≤ 0.001; and ****p ≤ 0.0001.

## 3 Results

### 3.1 Changes in rectal permeability with age during early development in rats

**Figure 1:**
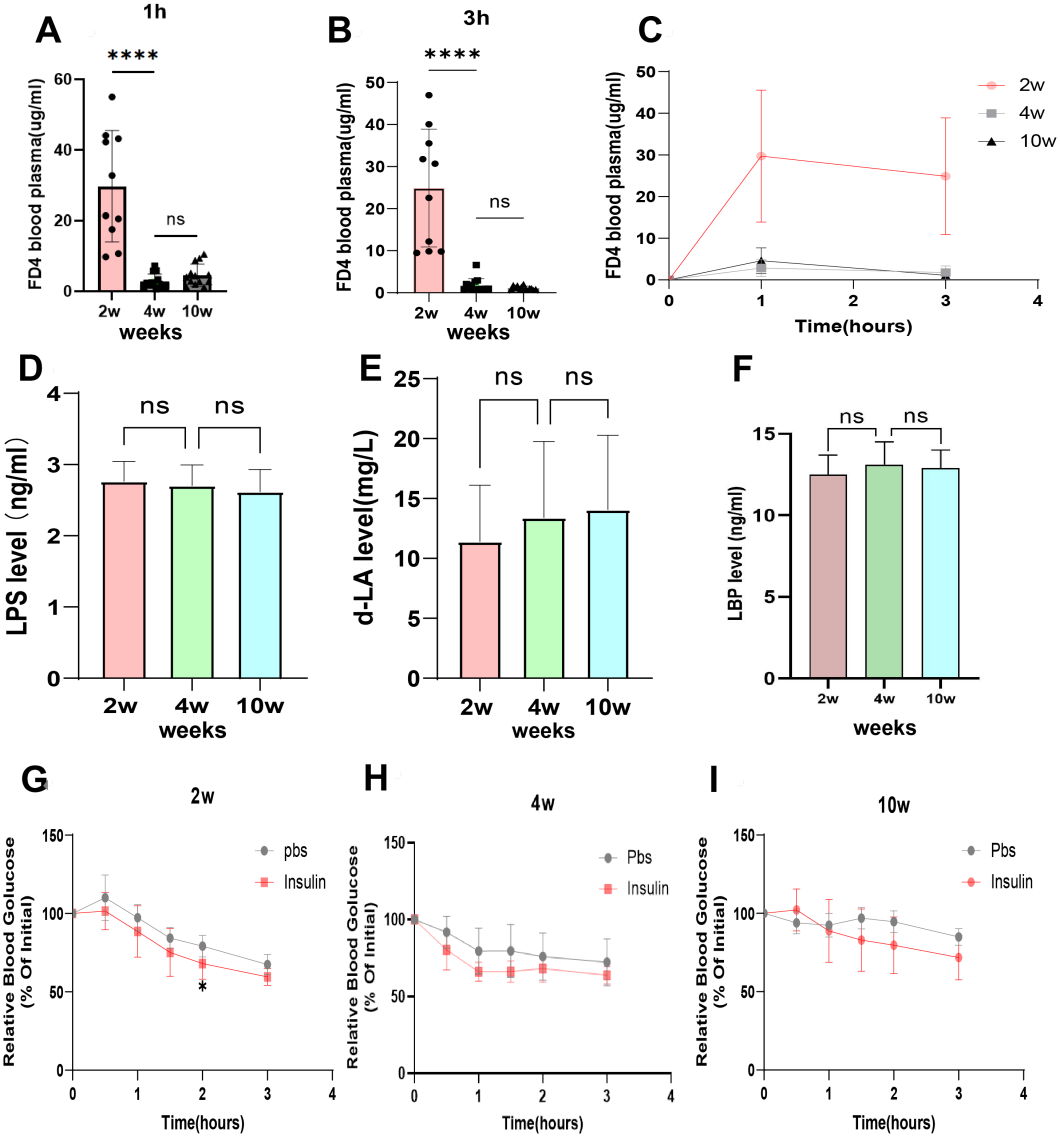
Changes in rectal permeability based on FITC - Dextran assay in rats at different ages. **(A,B)** The rectal permeability changes and plasma intestinal permeability markers at 1 h and 3 h, respectively. **(C)** The curve of change in FD4 concentration within 3 h of FITC-Dextran (FD4) retention enema. **(D–F)** The plasma intestinal permeability markers LPS, D-LA and LBP content, respectively. **(G–I)** The blood glucose levels after rectal administration of phosphate-buffered saline (PBS) or 5 IU/kg insulin in 2-week-old,4-week-old, and 10-week-old rats, respectively. *p ≤ 0.05; **p ≤ 0.01, ***p ≤ 0.001, and ****p ≤ 0.0001.

Other literature indicates that there is a significant reduction in paracellular permeability during the postnatal weeks in young rats (Patel et al., 2012). However, it remains unclear how rectal permeability changes with age. To investigate these changes from early to adulthood, we evaluated rectal permeability by measuring plasma concentrations of FITC-Dextran (FD4) via retention enema. The results demonstrated that FD4 enters the circulation from the rectum, reaching a peak concentration at 1 h, followed by a decrease at 3 h (as shown in Figure 1C). Plasma FD4 concentrations were significantly higher during 2-week-old compared to both 4-week-old and 10-week-old, at both the 1-h and 3-h marks. Additionally, there was no significant difference in rectal permeability between 4-week-old and 10-week-old (depicted in Figures 1A,B). This suggests that at 2 weeks of age, rectal permeability is significantly higher than that observed in 4-week-old and 10-week-old. By 4 weeks of age, rectal permeability no longer differs from that of adults.

Plasma levels of LPS, D-LA and LBP (lipopolysaccharide-binding protein) are considered potential markers of increased intestinal permeability (Seethaler et al., 2021). We examined the plasma levels of LPS, D-LA and LBP from early development (2-week-old rats) through adulthood (10-week-old rats). The results indicated no statistically significant difference in plasma levels of LPS (Figure 1D), D-LA (Figure 1E) or LBP (Figure 1F) among the three age groups. This lack of variation may be attributed to the immature structure of the intestinal flora in young rats and the influence of breastfeeding. D-LA is primarily produced from glucose metabolism (Levitt and Levitt, 2020), and breast milk predominantly contains lactose with minimal glucose (Boudry et al., 2021). These findings collectively suggest that systemic biomarkers such as D-LA, LPS, and LBP may not reliably reflect localized changes in rectal permeability in healthy rats during early life, particularly when structural and functional maturation of the rectal barrier is ongoing.

Finally, to assess whether the high rectal permeability in 2-week-old rats facilitates the absorption of rectally administered insulin, we monitored blood glucose levels in rats that received rectal insulin (5.8 kDa). Our results showed that, compared to the PBS-administered group, the blood glucose levels in the rectal insulin group at 2 h were significantly reduced. Furthermore, rectal administration of insulin significantly lowered blood glucose levels in 2-week-old rats (Figure 1G) but was ineffective in 4-week-old rats or 10-week-old rats (Figures 1H,I).

### 3.2 Histological changes in the rectal mucosa of rats significantly change with increasing age

**Figure 2:**
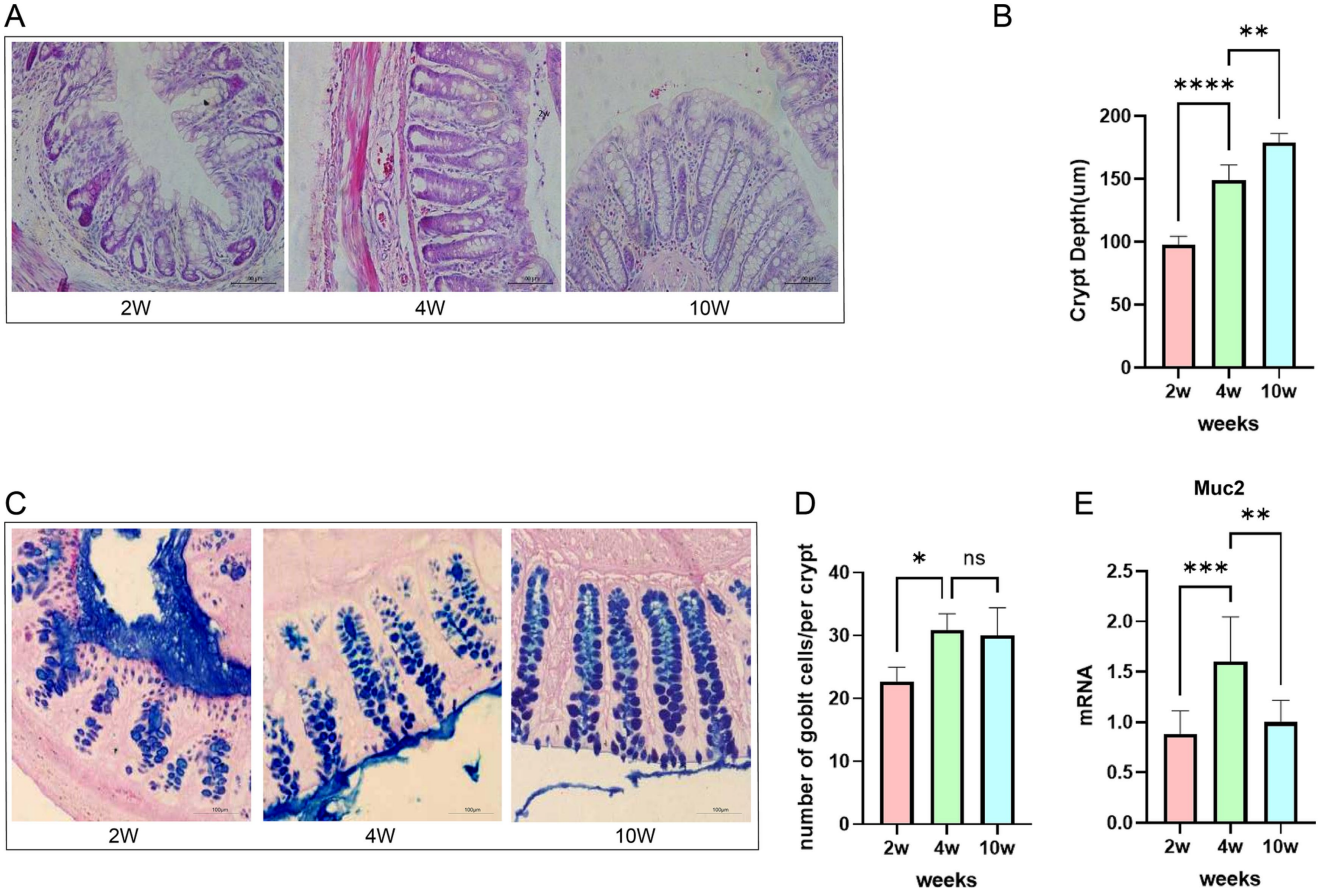
Microscopic images of histological sections of rat rectum stained with HE. **(A)** Age-related changes in rectal crypt depth, number of goblet cells, and Muc2 expression in rats. **(B)** Mean rectal crypt depth. **(C)** Microscopic images of histological sections of rat rectum stained with AB-PAS. **(D)** Mean number of goblet cells within each crypt. **(E)** Muc2 mRNA expression measured by RT-qPCR. *p ≤ 0.05; **p ≤ 0.01, ***p ≤ 0.001, and ****p ≤ 0.0001.

The rectum is the final section of the large intestine, characterized by a lack of villous structure and a distinct arrangement of intestinal crypts (Purohit et al., 2018). We performed hematoxylin and eosin (HE) staining and Alcian blue-periodic acid-Schiff (AB-PAS) staining on the rectal tissues of rats in three groups: 2-week-old,4-week-old, and 10-week-old, to analyze rectal histology. Our statistical analysis revealed a significant increase in the depth of the rectal crypts as rats progressed from young to adulthood, indicating age-related changes (Figure 2B). Notably, the rectal crypts of 2-week-old rats exhibited active proliferation, as illustrated in Figure 2C and indicated by the arrows in Figure 2A.

The surface of the rectal mucosa is coated with mucous layer, which not only lubricating the intestinal lumen but also serves as a barrier to protect against pathogenic bacteria and harmful substances entering the body (Capaldo et al., 2017). The primary component of this mucous layer is Muc2, which is secreted by goblet cells (Yang and Yu, 2021). To evaluate age-related changes in the rectal mucus barrier, we examined the number of goblet cells and the expression of mucin-2 mRNA. We observed a significant increase in the goblet cell density per crypt from 2 weeks of age in 2-week-old rats to 4 weeks of age in early developmental stages (Figure 2D). In addition, the expression of mucin-2 mRNA also showed a notable rise (Figure 2E), although the difference between 4 weeks and 10 weeks of age was not significant. These findings indicate that the development and maturation of the rectal mucus barrier occur rapidly during the early postnatal period (2–4 weeks of age), particularly when structural and functional maturation of the rectal barrier is ongoing.

### 3.3 Barrier functional structures tight junction protein expression and localisation changes with age

**Figure 3:**
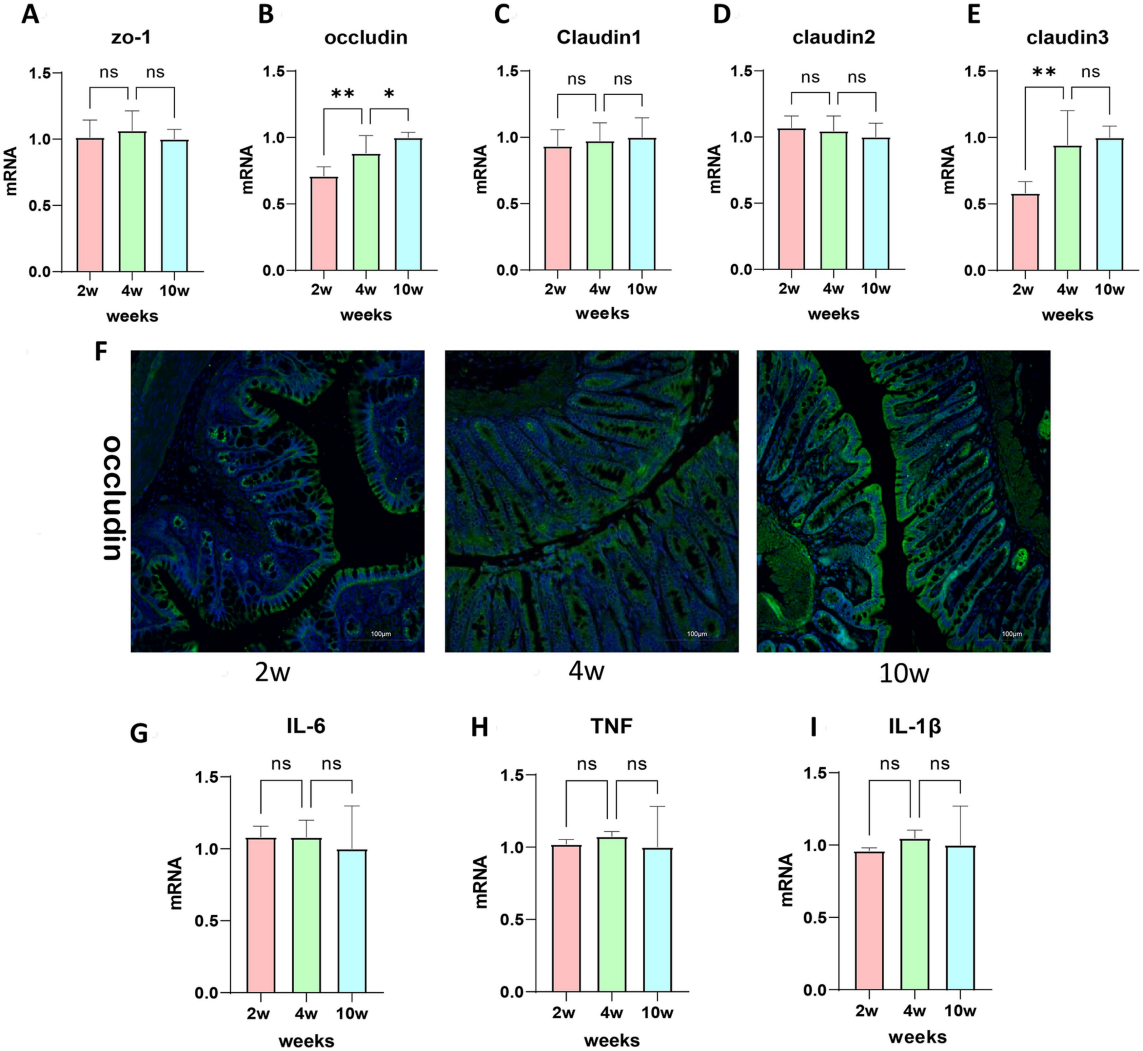
Developmental dynamics of rectal barrier components in postnatal rats. **(A–E)** ZO-1, Occludin, Claudin-1, Claudin-2, Claudin-3 mRNA expression values of different rectal tight junction proteins were detected using RT-PCR. **(F)** Immunofluorescence staining of rectal tight junction protein occludin. **(G–I)** IL-6, TNF, IL-1*β*mRNA levels of inflammatory factors using RT-PCR. *p ≤ 0.05; **p ≤ 0.01, ***p ≤ 0.001, and ****p ≤ 0.0001.

Tight junction proteins play a crucial role in controlling the pathways between intestinal epithelial cells, thereby regulating the permeability of various molecules (Hollander and Kaunitz, 2020). To investigate the differences in rectal permeability between young rats and adult rats, we assessed the gene expression and protein localization of different tight junction proteins in rectal tissue. Our study revealed that the expression levels of tight junction proteins ZO-1 (Figure 3A), Claudin-1 (Figure 3C), and Claudin-2 (Figure 3D) remained consistent across different ages. However, Occludin levels increased significantly with age (Figure 3B). Claudin-3 demonstrated a marked increase from 2 to 4 weeks of age and maintained stable levels through adulthood up to 10 weeks of age (Figure 3E). These proteins contribute significantly to the composition of the intestinal barrier, and a decrease in Occludin and Claudin-3 gene expression is associated with increased intestinal permeability. Immunofluorescence staining allowed us to localize Occludin, revealing that it was only partially expressed in the upper regions and crypts of the rectal tissue at 2 weeks of age in young children. In contrast, Occludin protein was more abundant and uniformly distributed throughout the crypts in 10-week-old rat tissues (Figure 3F). In 2-week-old rat tissues, occludin was predominantly expressed at the base of the crypts. This distribution pattern may be attributed to the presence of intestinal stem cells in the crypts, which differentiate into enterocytes and are more prevalent and active at the base of the crypts in 2-week-old and 4-week-old rats.

Additionally, we explored the effect of inflammatory cytokines on intestinal mucosal homeostasis and permeability. We examined the gene expression of IL-6, TNF-*α*, and IL-1β in rectal tissues and found no statistically significant differences in the mRNA expression levels of IL-6 (Figure 3G), TNF-α (Figure 3H), and IL-1β (Figure 3I) among rats at 2-, 4-, and 10-weeks of age (one-way ANOVA, *p* > 0.05).

### 3.4 Temporal changes in rectal microbial communities during early development in rats

**Figure 4:**
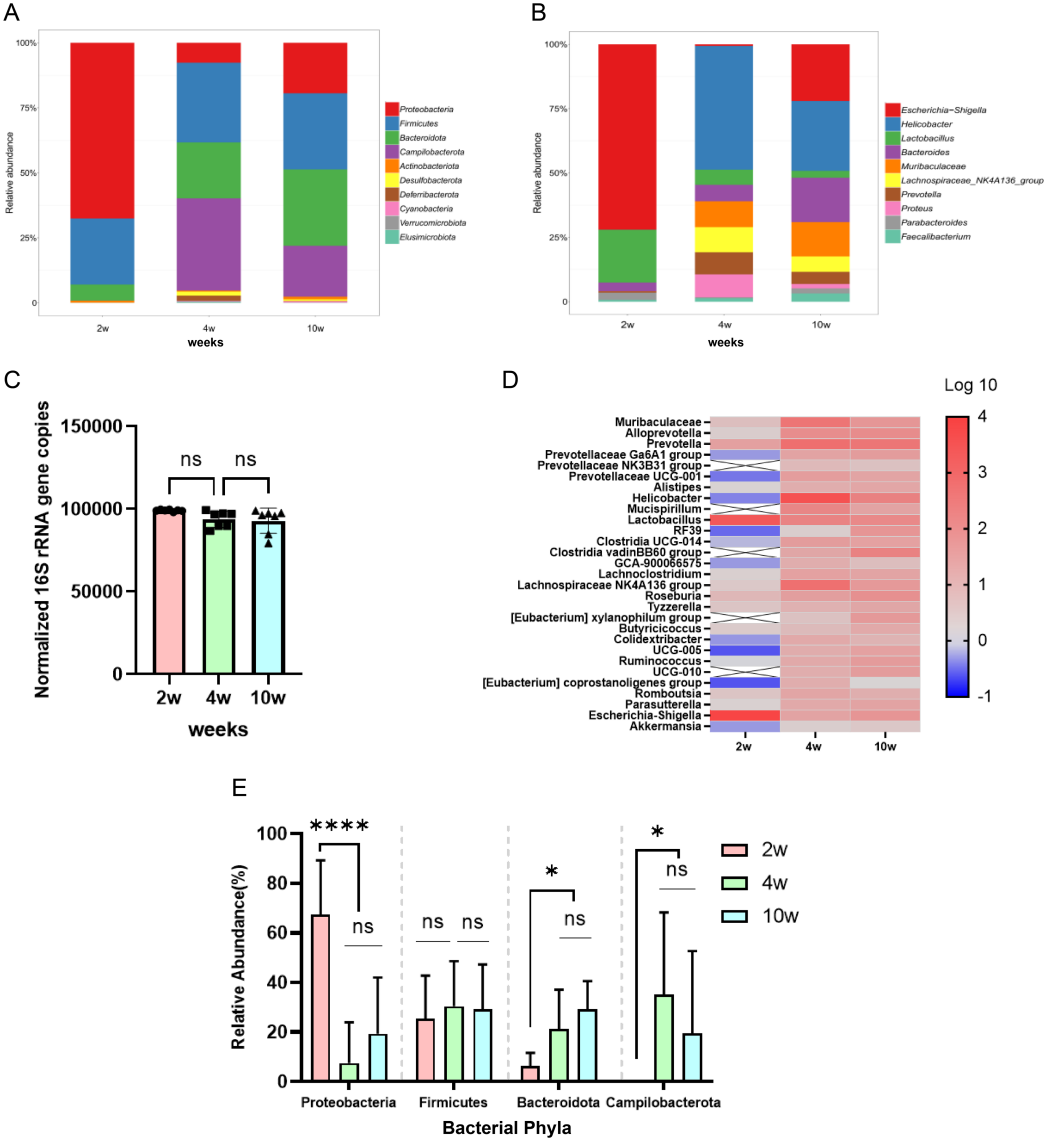
Age-dependent changes in the structure of the rectal microbiota of rats during early development. **(A)** Stacked bars representing the relative abundance of bacteria at the ‘phylum’ classification level. **(B)** Stacked bars of the relative abundance of bacteria at the ‘genus’ classification level. **(C)** Total bacterial counts of the rectal microbiota. **(D)** Heatmap of changes in the abundance of differentially abundant organisms at the genus level in the rectal microbiota. **(E)** Changes in the relative abundance of the major bacterial phyla. *p ≤ 0.05; **p ≤ 0.01, ***p ≤ 0.001, and ****p ≤ 0.0001. (*n* = 7 biological replicates per group; log10 denotes genus-level species relative abundance data multiplied by 10,000 and then log10-transformed).

Previous reports have shown that there are age-dependent changes in the host gut microbiota after birth, with variations in the diversity and composition of gut flora across different intestinal segments. Currently, microbiota analyses are primarily conducted using fecal samples, which do not provide information specific to certain intestinal segments. The rectum, located at the end of the gastrointestinal tract and close to the body surface, has not been extensively studied regarding its microbiota. To comprehensively characterize the age-dependent shifts in rectal microbiota composition, we analyzed bacterial communities at both the phylum and genus levels. Figure 4A illustrates the relative abundance of major bacterial phyla across the three age groups. At 2 weeks of age, Proteobacteria dominated the rectal microbiota (relative abundance >60%), followed by Firmicutes (>25%). By 4 weeks of age, the relative abundance of Proteobacteria significantly decreased to <20% (*p* < 0.0001), while Firmicutes and Bacteroidetes became predominant. This transition suggests a maturation of the microbial community structure toward a more adult-like profile.

At the genus level (Figure 4B), Escherichia-Shigella (Proteobacteria) and Lactobacillus (Firmicutes) were highly abundant in 2-week-old rats. In contrast, 4- and 10-week-old rats exhibited increased proportions of Muribaculaceae (Bacteroidetes) and Akkermansia (Verrucomicrobia), genera known for their roles in mucosal barrier modulation.

Our observations further indicated no significant changes in total bacterial counts from 2 to 10 weeks of age (Figure 4C). Heatmap analysis (Figure 4D) revealed distinct clustering of microbial genera by age, with 4- and 10-week-old groups showing higher similarity compared to 2-week-old rats. These findings collectively highlight dynamic remodeling of the rectal microbiota during early development. Furthermore, we found significant similarities between the rectal microbiota at 4 weeks and 10 weeks of age (Figure 4E).

### 3.5 Spatiotemporal dynamics of rectal microbiota and barrier function during postnatal development in rats

**Figure 5:**
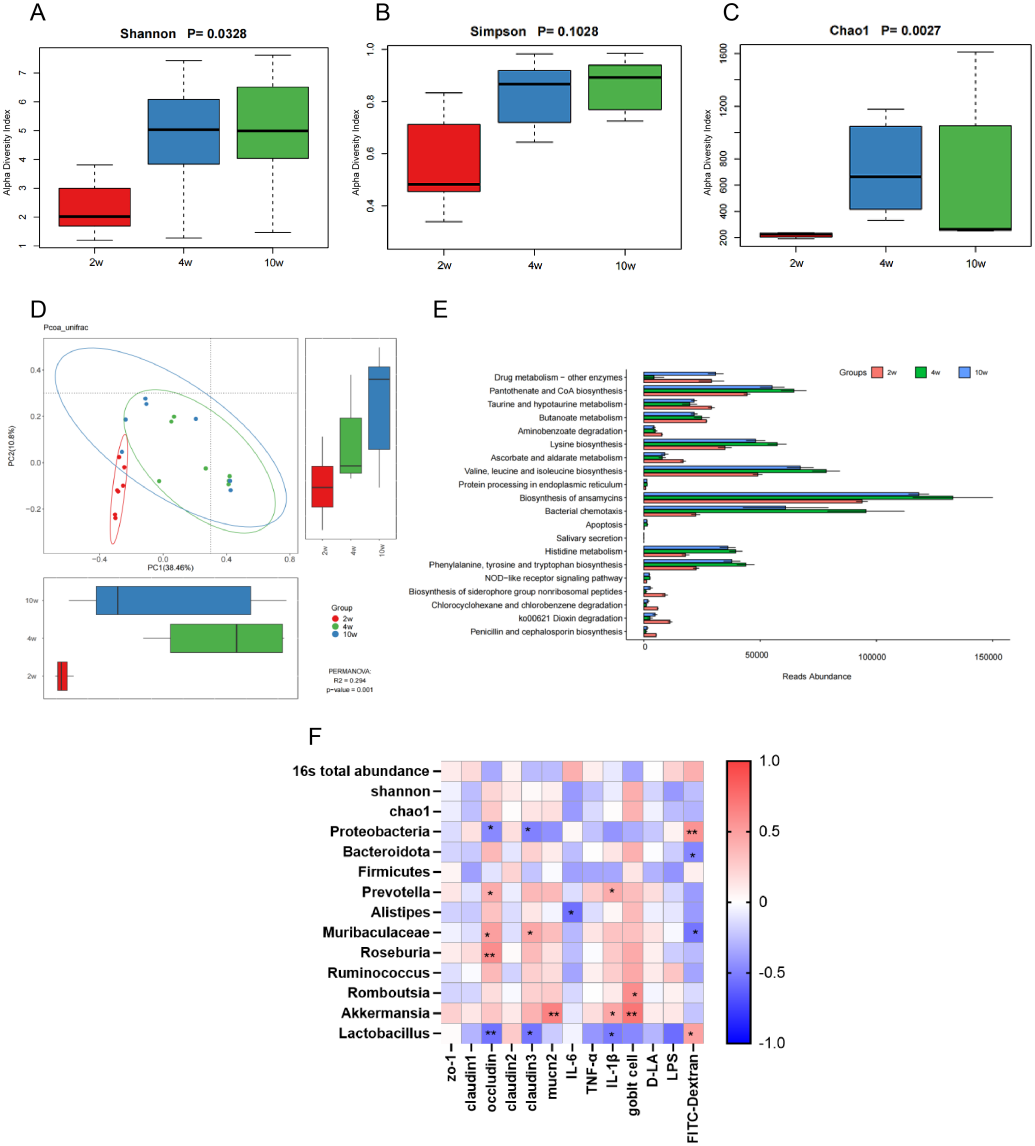
Diversity and functional analysis of temporal changes in the rectal microbiota during the early development stage of rats. **(A)** The *α*-diversity indices of the rat rectal microbiota include Shannon’s index. **(B,C)** Simpson’s index and species homogeneity calculated by the Chao1 index measure. **(D)** The β-diversity of rectal microbes is constructed using the Unweighted Unifrac phase dissimilarity index for principal coordinates analysis (PCoA). **(E)** PICRUSt functional prediction of KEGG metabolic pathway abundance and enrichment is based on 16S rDNA sequences for PICRUSt functional prediction of KEGG metabolic pathway abundance enrichment. **(F)** Correlation analysis is conducted between the gut microbial phylum, α-diversity, total bacterial abundance, barrier structure, and gene expression. *p ≤ 0.05; **p ≤ 0.01, ***p ≤ 0.001, and ****p ≤ 0.0001.

We studied the changes in gut microbial abundance and community profiles during the early development of rats, focusing specifically on the diversity of the rectal microbiota. Statistical analyses using Shannon’s index (*p* < 0.05) (Figure 5A), Simpson’s index (*p* = 0.1028) (Figure 5B), and the Chao1 index (*p* < 0.01) (Figure 5C) indicated that while Shannon (diversity) and Chao1 (richness) indices showed significant age-dependent changes, Simpson’s index (evenness) did not reach statistical significance (*p* > 0.05), suggesting that microbial community evenness remained relatively stable during early development. Additionally, we analyzed the differences in *β*-diversity using the Unweighted Unifrac phase dissimilarity index to construct principal coordinate analysis (PCoA). It was clear along the PC1 axis that the principal coordinate regions of the two groups (4-week-old and 10-week-old rats) were highly overlapping, whereas the 2-week-old group was distinct from them. Furthermore, the three age groups were approximately divided into two regions (variance = 38.46%) according to PERMANOVA (R-squared: 0.294; *p* = 0.001). In the PC2 direction, significant differences in rectal microbiota between the 2-week-old, 4-week-old, and 10-week-old rats were observed (variance = 20.2%) (Figure 5D). Recent studies have revealed that the gut microbiota and its metabolites can regulate the transcriptome profile, proliferation, and differentiation of intestinal epithelial cells (IECs), thereby maintaining the integrity of the intestinal barrier. Therefore, we predicted the enrichment of KEGG metabolic pathway abundance using PICRUSt function (Figure 5E). We found that from young (2 weeks of age) to adulthood, several metabolic pathways were downregulated, including iron-carrier group non-ribosomal peptide biosynthesis, chlorocyclohexane and chlorobenzene degradation, dioxin degradation, and penicillin and cephalosporin biosynthesis. Meanwhile, pathways such as lysine biosynthesis, valine, leucine, and isoleucine biosynthesis, bacterial chemotaxis, histidine metabolism, and the biosynthesis of phenylalanine, tyrosine, and tryptophan were upregulated.

Finally, we assessed the association between the intestinal mRNA gene expression of various tight junction proteins (such as Claudin-1, ZO-1, and Occludin), markers of intestinal permeability (including LPS, D-LA, and LBP), indicators of rectal permeability (measured by FITC-Dextran plasma concentration), mRNA expression of inflammatory factors (IL-6, TNF-*α*, IL-1β), the number of Goblet cells, Muc2 mRNA expression, and the development of rectal microbiota (including intestinal microbiota portal, α-diversity, and total bacterial abundance) by plotting Spearman correlation analysis (R) heatmaps (*p* < 0.01, *p* < 0.05) (Figure 5F).

Based on our analyses, we discovered several correlations of interest. The expression of the tight junction protein Claudin-1 was significantly negatively correlated with total bacterial abundance (16S total abundance) (Spearman’s *R* = −0.495, *p* = 0.023). The expression of Occludin showed a significant negative correlation with the abundance of the phylum Degenerobacteria (Spearman’s *R* = −0.464, *p* = 0.034) and the abundance of Lactobacillus (Spearman’s *R* = −0.553, *p* = 0.009). Conversely, it was significantly positively correlated with the abundance of Muribaculaceae bacteria (Spearman’s *R* = 0.503, *p* = 0.020). The expression of Claudin-3 was significantly negatively correlated with the abundance of *Saccharomyces cerevisiae* (Spearman’s *R* = −0.497, *p* = 0.022) and Lactobacillus (Spearman’s *R* = −0.542, *p* = 0.011), while it showed a significant positive correlation with Muribaculaceae (Spearman’s *R* = 0.456, *p* = 0.038). Furthermore, mucin Muc2 expression was significantly positively correlated with the abundance of Akkermansia (Spearman’s *R* = 0.657, *p* = 0.001). The expression of the inflammatory factor IL-1β was significantly negatively correlated with Lactobacillus (Spearman’s *R* = −0.495, *p* = 0.015) and positively correlated with Akkermansia (Spearman’s *R* = 0.495, *p* = 0.034) as well as Prevotella (Spearman’s *R* = 0.437, *p* = 0.048). The number of goblet cells was significantly positively correlated with Romboutsia (Spearman’s *R* = 0.595, *p* = 0.046) and Akkermansia (Spearman’s *R* = 0.716, *p* = 0.011). Additionally, the plasma concentration of FITC-Dextran exhibited a significant negative correlation with Bacteroides (Spearman’s *R* = −0.488, *p* = 0.025) and Muribaculaceae (Spearman’s *R* = −0.616, *p* = 0.003), while showing a significant positive correlation with Proteobacteria (Spearman’s *R* = 0.565, *p* = 0.008) and Lactobacillus (Spearman’s *R* = 0.490, *p* = 0.024).

In summary, the expression levels of the tight junction protein Occludin were significantly positively correlated with Muribaculaceae, Prevotella, and Roseburia. The mucin Muc2 transcript levels were significantly positively correlated with Akkermansia. The number of Goblet cells showed a significant positive correlation with both Romboutsia and Akkermansia. Importantly, rectal permeability, as measured by FITC-Dextran plasma concentration, exhibited a negative correlation with Muribaculaceae and Bacteroides. Moreover, the abundances of Muribaculaceae, Mucispirillum, and Akkermansia bacteria in the rectum were significantly higher at 4 weeks compared to 2 weeks. This suggests that the high rectal permeability observed at 2 weeks may be linked to the immaturity of the rectal microbiota, which impacts barrier function.

## 4 Discussion

The human gastrointestinal tract houses hundreds of millions of microorganisms that have developed a mutually beneficial and cooperative relationship with humans over a long period of co-evolution. The human body provides a stable and nutrient-rich environment for these microorganisms through the diet while simultaneously fulfilling its nutritional needs. Intestinal microorganisms assist in maintaining and repairing various intestinal functions, such as crypt depth, the proliferation of intestinal epithelial cells, vascular density, and mucus thickness. They also play a crucial role in the development of intestinal immunity and the synthesis of nutrients, including vitamins and serotonin (Tremaroli and Bäckhed, 2012). Furthermore, the microbiota in the intestines mainly produces SCFAs by fermenting intestinal contents. SCFAs support the growth of beneficial bacteria, inhibit the colonization of pathogenic bacteria, regulate the expression of proteins related to tight junctions, and maintain the intestinal barrier function (Mariadason et al., 1997). Research has shown that butyrate can increase the expression of the transcript levels of Occludin and ZO-1 in IPEC-J2 cells and reduce intestinal permeability in rat. Additionally, Tong et al. found that propionate can enhance the expression of tight junction proteins, such as Occludin and ZO-1, along with calcineurin. Incorporating foods rich in SCFAs into the diets of human subjects can elevate the expression of mucins Muc2 and Occludin in the colon (Hald, 2015). Currently, the study of intestinal flora is primarily conducted through fecal sampling. In this context, we focused on collecting samples from the rectal area to investigate the relationship between intestinal microbiota and rectal barrier function.

Beneficial bacteria such as Muribaculaceae, Prevotella, and Roseburia generate short-chain fatty acids from food residues in the intestinal tract. Their main products include propionate (Smith et al., 2019), acetate (Iljazovic et al., 2021), and butyrate (Nie et al., 2021), respectively. Our findings revealed a significant positive correlation between Occludin expression and the presence of Muribaculaceae, Prevotella, and Roseburia. This suggests that these bacteria in rats can promote the expression of the rectal epithelial tight junction protein Occludin, thereby enhancing the maturation of the rectal barrier structure. This observation is supported by Tian et al., who demonstrated that short-chain fatty acids increased the expression levels of intestinal tight junction mRNAs and proteins, reduced intestinal permeability, and improved intestinal barrier function in rats (Tian et al., 2021). Similar conclusions have been reported in other studies, such as a study showing that adding sodium butyrate to the diets of weaned piglets improved intestinal morphology, increased jejunal trans-epithelial resistance, decreased the ectopic entry of FD4 into the bloodstream (a measure of intestinal barrier integrity), and enhanced overall intestinal barrier function in these piglets (Huang et al., 2015).

An increase in Proteobacteria has been suggested as a potential diagnostic feature of ecological dysbiosis in the intestinal flora and associated disease risk (Shin et al., 2015). This is due to its potential to induce nonspecific inflammation in the intestines, leading to damage to the intestinal barrier structure (Hiippala et al., 2018). Elevated levels of Proteobacteria are often found in various intestinal diseases, such as irritable bowel syndrome, metabolic syndrome, and inflammatory bowel disease (Litvak et al., 2017). Our correlation analysis revealed a significant negative correlation between the expression of tight junction proteins, Occludin and Claudin-3, and Proteobacteria levels. Microbiota characterization of the rectal area showed that Proteobacteria had a relative abundance exceeding 60% during the 2-week-old rats and then dropped significantly to below 20% as the rats matured. This indicates the presence of Proteobacteria in the rectal area of rats during young and suggests that the immaturity of the rectal barrier function during this time coincides with the disruption of tight junction proteins caused by Proteobacteria.

Bacteroides are the most abundant Gram-negative bacteria in the gut, comprising more than 30 species capable of breaking down polysaccharides into oligosaccharides or monosaccharides. These byproducts serve as substrates for fermentation by other bacteria that produce short-chain fatty acids (Cheng et al., 2022). Research has shown that Bacteroides possess genes encoding enzymes that degrade and ferment different carbohydrates. Additionally, they engage in various biological interactions with other beneficial microorganisms to enhance gut function and health (Zhang et al., 2022). In our study, we found a significant negative correlation between the plasma concentration of FITC-Dextran (a marker for rectal permeability) and both Bacteroides and Muribaculaceae. This observation suggests that these bacteria can reduce rectal permeability, leading us to hypothesize that they may enhance barrier function through the production of short-chain fatty acids. Akkermansia is another important component of the intestinal microbiota, particularly involved in intestinal propionate metabolism and production. It has been shown to promote the maturation of the intestinal barrier by facilitating the formation of intestinal organoids and the proliferation of intestinal epithelial cells (Duan et al., 2023). In our study, we observed a positive correlation between the expression of mucin MUC2, the number of Goblet cells, and Akkermansia. Akkermansia increased the expression of Goblet cells in the rectal crypts; since mucus is primarily secreted by Goblet cells, this suggests that Akkermansia plays a significant role in enhancing the maturation of the rectal mucus barrier, consistent with previous findings (Shin et al., 2014). Furthermore, Akkermansia was found to increase the expression of tight junction protein ZO-1, Occludin, and the number of Goblet cells, promoting mucus production, reducing intestinal permeability, and enhancing epithelial barrier function (Grander et al., 2018; Huck et al., 2020)。.

In conclusion, this study found that the expression levels of Occludin, Claudin-3, and Muc2 transcripts increase with age. Beneficial bacteria, such as Akkermansia, which promotes mucus secretion, as well as Muribaculaceae, Prevotella, Roseburia, and Romboutsia, which produce short-chain fatty acids, were observed to have low abundance during the 2-week-old but increased in prevalence from 2-week-old to 10-week-old. The expression level of Occludin was positively correlated with Muribaculaceae, Prevotella, and Roseburia, while Muc2 expression was positively correlated with Akkermansia. Additionally, the number of goblet cells showed a positive correlation with Romboutsia and Akkermansia, and FITC-Dextran plasma concentration was negatively correlated with Muribaculaceae and Bacteroides. These findings suggest that changes in rectal barrier function are significantly linked to the composition of gut microbiota. It is hypothesized that gut microbiota metabolites, specifically short-chain fatty acids, play a role in promoting the expression of rectal tight junction proteins, such as Occludin and Claudin-3. This, in turn, enhances rectal barrier function and normalizes rectal permeability.

## 5 Conclusion

This study explored the relationship between rectal permeability and the microorganisms present in the intestinal flora of SD rats. The findings revealed a significant correlation between changes in rectal permeability and the composition of microbial flora in young rats. It is hypothesized that the gradual decrease in physiological rectal permeability with age in rats may be attributed to increased expression of rectal tight junction proteins, specifically Occludin and Claudin-3. Additionally, metabolites produced by intestinal microbiota, such as short-chain fatty acids, may enhance rectal barrier function, thereby reducing rectal permeability to a low normal level. In conclusion, this study offers new insights into how the microbial composition of the rectal microbiota evolves from young to adulthood, influencing rectal barrier function and structure. These findings could improve our understanding of rectal drug delivery in children.

## Data Availability

The raw reads of the 16S rDNA sequence data were deposited into the NCBI Sequence Read Archive (SRA) database under BioProject accession number PRJNA1243059.
